# Multipolar adaptive traction makes endoscopic submucosal dissection feasible for large neoplastic area of the fundus in patient with familial adenomatous polyposis

**DOI:** 10.1055/a-2135-8682

**Published:** 2023-08-21

**Authors:** Elena De Cristofaro, Louis-Jean Masgnaux, Jean-Christophe Saurin, Jérémie Jacques, Bertrand Napoléon, Jérôme Rivory, Mathieu Pioche

**Affiliations:** 1 Gastroenterology Unit, Department of Systems Medicine, University of Rome Tor Vergata, Rome, Italy; 2 Gastroenterology and Endoscopy Unit, Edouard Herriot Hospital, Hospices Civils de Lyon, Lyon, France; 3 Gastroenterology and Endoscopy Unit, Dupuytren University Hospital, Limoges, France; 4 Gastroenterology and Endoscopy Unit, Mermoz Hospital, Lyon, France

Multiple fundic gland polyps (FGPs) are often found in patients with familial adenomatous polyposis (FAP) but are usually benign. FAP-associated dysplasia is rare but, where found, is fundic, whitish, and flat 1 . Endoscopic submucosal dissection (ESD) is a good but challenging option for achieving complete resection because the top of the fundus is difficult to reach and because the multiple FGPs obscure the margins of the dysplastic area, making incision difficult. Dedicated scopes such as the double bending therapeutic gastroscope (2TQ260M; Olympus, Tokyo, Japan) have been designed for fundic lesions, but are not available in Europe 2 . Therefore, alternative strategies are much needed. Adaptive traction devices are useful for complex lesions 3 4 5 . We describe an option for ESD of difficult fundic lesions using the 4-point multipolar adaptive traction device (A-TRACT 4).

We report the case of a 42-year-old patient with FAP and a large, whitish, dysplastic area in the fundus ( Fig. 1 ). The lesion was anatomically difficult to reach, but by pushing a long loop of the scope against the antrum, the fundus was reached with a relatively tangential approach to the lesion. After circumferential incision and trimming, the four loops of the A-TRACT 4 device were fixed to the lateral edges of the lesion. The rubber band was fixed by another clip to the opposite wall ( Fig. 2 ). The dissection was started with appropriate traction. After cutting three-quarters of the lesion, proper traction was re-established by tightening the A-TRACT 4. The procedure was completed with optimal exposure of the submucosa ( Video 1 ). This technique allowed a curative R0 resection of the 9 × 8 cm lesion in 110 minutes.

**Fig. 1:**
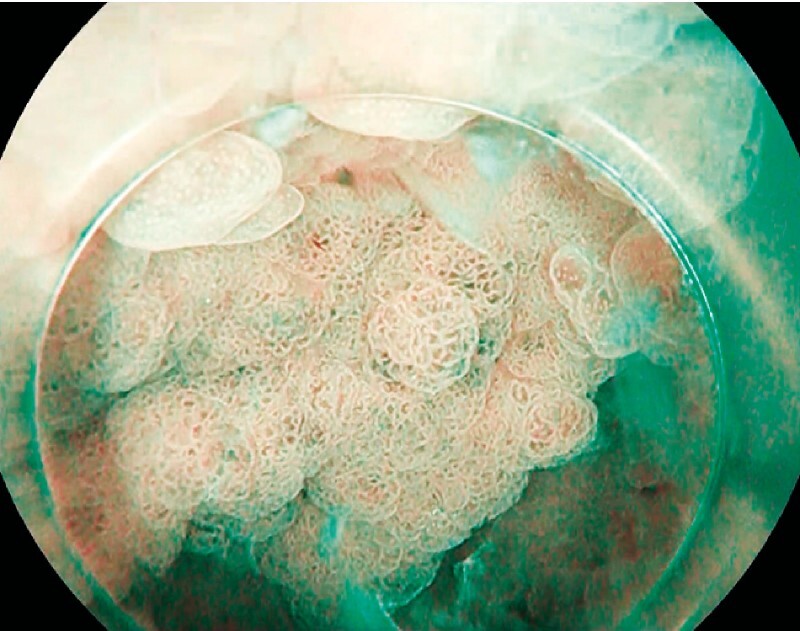
Large fundic lesion in a patient with familial adenomatous polyposis.

**Fig. 2:**
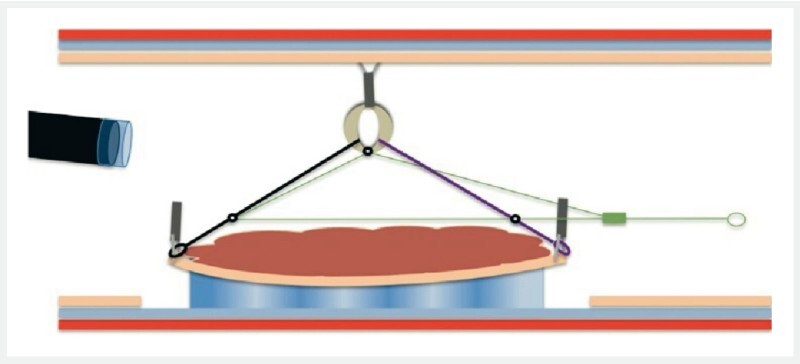
Schematic representation after application of A-TRACT 4, allowing excellent exposure of the submucosa.

**Video 1** Challenging endoscopic submucosal dissection of a large fundic lesion using A-TRACT 4 in a patient with familial adenomatous polyposis.


We hypothesize that such a dedicated device could facilitate ESD, with the advantage of allowing traction to be adapted during the procedure to change the conformation of the fundus for optimal submucosal exposure. Furthermore, the combined use of additional endoscope looping in the antrum could be helpful in resection of fundic lesions.

Endoscopy_UCTN_Code_TTT_1AO_2AG

## Notes

Endoscopy E-Videos

https://eref.thieme.de/e-videos
*E-Videos* is an open access online section of the journal *Endoscopy* , reporting on interesting cases and new techniques in gastroenterological endoscopy. All papers include a high-quality video and are published with a Creative Commons CC-BY license. Endoscopy E-Videos qualify for HINARI discounts and waivers and eligibility is automatically checked during the submission process. We grant 100% waivers to articles whose corresponding authors are based in Group A countries and 50% waivers to those who are based in Group B countries as classified by Research4Life (see: https://www.research4life.org/access/eligibility/ ). This section has its own submission website at https://mc.manuscriptcentral.com/e-videos
